# Reliability and validity of the Chinese version of the Walsh Family Resilience Questionnaire among community-dwelling disabled elderly individuals (WFRQ-CE)

**DOI:** 10.3389/fpsyg.2022.1095958

**Published:** 2023-01-17

**Authors:** Xiangchun Zhang, Anni Wang, Tingyu Guan, Yi Kuang, Yuyi Zhang, Fangqi Wu

**Affiliations:** School of Nursing, Fudan University, Shanghai, China

**Keywords:** disabled elderly, family resilience, questionnaire, reliability, validity

## Abstract

**Objective:**

To test the reliability and validity of the Chinese version of the Walsh Family Resilience Questionnaire among community-dwelling disabled elderly individuals (WFRQ-CE).

**Methods:**

Convenience sampling was used to select 566 dyads of disabled elderly individuals and their caregivers. The Walsh Family Resilience Questionnaire Chinese Version (WFRQ-C) was tested among elderly individuals. The Family Care Capacity Scale for Elderly Patients (FCCSE) was used as a concurrent validation tool for the caregivers, and the Psychological Resilience Scale (CD-RISC-10), and the Social Support Assessment (SSRS-10) were used as concurrent validation tools for both the elderly individuals and the caregivers.

**Results:**

Exploratory factor analysis (EFA) revealed four common factors–“Family belief,” “Organization and problem solving,” “Family communication,” and “Utilization of external resources”–with a cumulative variance contribution rate of 56.94%. Confirmatory factor analysis (CFA) yielded the following fit indices: chi-square/freedom degree (χ2/df) = 2.007, Tucker Lewis index (TLI) = 0.900, incremental fit index (IFI) = 0.917, comparative fit index (CFI) = 0.916, parsimony goodness-of-fit index (PGFI) = 0.681, and root-mean-square error of approximation (RMSEA) = 0.060. The concurrent scales were significantly correlated with the WFRQ-C total score and the scores for each factor (*r* values between 0.23 and 0.60, *P* < 0.01). The Cronbach’s alpha coefficient was 0.93 for the WFRQ-CE and 0.87, 0.83, 0.89, and 0.65 for the four factors; the retest reliability was 0.96 for the total scale and 0.95, 0.92, 0.92, and 0.95 for the four factors; the split-half reliability was 0.85 for the total scale, and 0.81, 0.78, 0.79, and 0.68 for the four factors.

**Conclusion:**

The WFRQ-CE has good reliability and validity among community-dwelling disabled elderly individuals and can be used to evaluate the level of family resilience.

## 1. Introduction

In 2020, 190.64 million people in China were aged above 65 years old, accounting for 13.5% of the total population of the country (Office of the Leading Group of the State Council for the Seventh National Population Census, 2021). Aging often coexists with disability (O’Young et al., 2019). Disability, which refers to the loss or limitation of a person’s main activities or living ability in daily life, is an important indicator of individual health (Wenjuan and Meng, 2015). According to the data from the ‘‘Fourth National Sampling Survey of the Living Conditions of the Elderly in Urban and Rural Areas of China’’ in 2016, there were approximately 40.63 million disabled and semi-disabled elderly people in China, an increase of 7.63 million compared with the end of 2010, accounting for 18.3% of the elderly population^1^. It is estimated that the proportion of disabled elderly individuals in the total population will increase from 1.15% in 2015 to 3.1% in 2054 (Yuejun et al., 2017). The most prevalent disabilities are physical disabilities, with hearing loss having a prevalence of 8.3%, physical disability having a prevalence of 6.1%, and visual disability having a prevalence of 4.6% among elderly individuals in China (O’Young et al., 2019). The positive association between the prevalence of disability and age reflects an accumulation of health risks, including chronic illness and injuries. On the one hand, with the accelerated process of aging, the cost and demand of care are increasing for disabled elderly individuals (Zeng et al., 2015; O’Young et al., 2019). On the other hand, weaker social mobility and the ability to access social resources make it difficult for disabled elderly individuals to meet their health needs (Wu et al., 2021). Influenced by the social culture and environment, approximately 90% of disabled elderly individuals in China are cared for by their families, and the form of socialized care is less at present (Qun et al., 2015; Xurong and Liping, 2016). To try to meet the needs of care, the family plays an important role in this process (Wu et al., 2021).

Family resilience refers to the ability of a family to maintain balance during a crisis, and some families have the ability to recover from adversity and stress and gain new strengths and social resources (Hawley and DeHaan, 1996; Walsh, 2003). Walsh’s theoretical model of family resilience, including family belief systems, patterns, and communication, illustrated the beliefs and coping ability of family members, the process by which families use resources from within and outside the family to cope with adversity (Walsh, 2003). Through family resilience theory, researchers can determine the process by which family members develop and exhibit family resilience when their families are undergoing changes and crises. Family resilience theory is widely studied in the context of families of cancer patients (Li et al., 2019; Chen et al., 2021), families of children with chronic diseases (Dong et al., 2021; Zhengqiao and LuoXiuzhuang., 2022), families of patients with certain diseases (Teahan et al., 2018) and families of certain occupations. Currently, research on disabled elderly individuals mainly focuses on the personal resilience of family caregivers (Koerner and Shirai, 2019) and lacks an exploration of the overall process of the family in coping with stress. A majority of families face challenges of insufficient care capability in meeting the care needs of elderly individuals, and it is necessary to study family resilience and the process of stress adaptation in the family unit of disabled elderly individuals and their caregivers (Roberto and Blieszner, 2015).

One of the first steps of research is evaluating relevant variables, and thus, it is necessary to find appropriate evaluation tools. In the field of healthcare, some scholars recommend the 54-item Family Resilience Assessment Scale (FRAS) developed by Sixbey (2005) and the 32-item WFRQ (Zhou et al., 2020), both of which are based on Walsh’s theoretical model of family resilience, which was developed by Walsh based on clinical work experience and an extensive review of the literature in 2002 (Walsh, 2002). Three scholars have translated and introduced the FRAS in China, thus yielding two assessment tools for families of cancer patients (Li et al., 2016; Yingwe et al., 2017) and one for families of children with chronic diseases (Chaoqun et al., 2018). The applicability of these versions for the families of disabled elderly people needs to be further studied, and some versions of the FRAS include too many items, which can lead to response burden among respondents. The WFRQ consists of 32 items across three main factors: family belief system (13 items), family organizational processes (9 items), and communication, and problem-solving processes (10 items). Subsequently, Italian scholars (Rocchi et al., 2017) examined the family resilience of 421 patients with chronic diseases and obtained a model with 26 items in three domains: shared beliefs and support, family organization and interaction, and utilization of social resources. Iranian scholars (Dadashi Haji et al., 2018) translated and tested the WFRQ-IT among 350 adolescents. Sabah et al. (2021) examined the WFRQ in a sample of 380 individuals from Iraqi and Algerian families, and Duncan et al. (2020) examined the WFRQ in a sample of 603 university students in the United States. Polish scholars (Nadrowska et al., 2022) tested and revised a model of 31 items across three factors among 930 Poles. Chinese scholars Li and Li (2021) examined the Italian version of the WFRQ (WFRQ-IT) among 716 stroke survivors and their caregivers. The items and internal structure of the WFRQ will change and differ in different stress–stressed groups and cultural backgrounds. There has been no exploration or verification of the WFRQ among disabled elderly individuals.

The family pressure and care problems of community-dwelling disabled elderly individuals have become increasingly prominent (Min and Weiliang, 2022). The impact of family resilience as a positive force is an important basis for future interventions, and a reasonable and stable family resilience assessment tool is an important prerequisite. Previously, our research team recently translated and revised the Walsh Family Resilience Questionnaire Chinese Version (WFRQ-C), which has 26 items across three factors, among 800 adult community residents by combining the Classical Theory Test (CCT) and Item Response Theory (IRT). The WFRQ-C has been shown to have good reliability and validity among community adult residents (Wang and Lu, 2022). Based on the existing preliminary research, this study aims to test the applicability of the WFRQ-C in community-dwelling disabled elderly individuals to lay the foundation for providing measurement tools for future related survey and intervention studies.

## 2. Materials and methods

### 2.1. Participants

Using the convenience sampling method, recruitment was carried out in multiple centers in China, including Shanghai, Anhui, Gansu, Sichuan, Chongqing, Guizhou, and other places. By contacting community workers, investigators visited surrounding communities from January 2021 to March 2022 to recruit disabled elderly individuals and their caregivers who met the inclusion criteria and who were willing to participate in the study.

The inclusion criteria for disabled elderly individuals were as follows: (1) age ≥ 60 years old and classified as mildly or more severely physically disabled by the Activities of Daily Living scale; (2) primarily received home care; (3) able to communicate verbally; and (4) understood the purpose of the research and participated in the research voluntarily. The exclusion criteria were as follows: (1) serious physical diseases or extreme weakness; and (2) a mental disorder or cognitive impairment.

The inclusion criteria for primary caregivers were as follows: (1) age ≥ 18 years old; (2) responsible for the primary care of elderly family members; if there are several caregivers, the person who cared for the elderly individual for the longest time was chosen; (3) cared for the elderly individual for at least 1 month; (4) clear awareness, normal communication, and understanding skills; and (5) understood the purpose of the research and volunteered to participate in the research. The exclusion criteria were as follows: (1) mental and cognitive impairments; and (2) formal caregivers who received financial compensation.

The sample size was determined using a subject-to-item ratio of 5–10:1 (Lu et al., 2015), based on the WFRQ-C of 26 items. A sample size of 260 to 520 was determined for exploratory factor analysis (EFA) and confirmatory factor analysis (CFA). Forty elderly individuals were selected and completed the questionnaire again after 2 weeks to evaluate the retest reliability. A total of 607 questionnaires were distributed, and 566 valid questionnaires were recovered (mainly distributed in five provinces across China: Shanghai 24.4%, Anhui 27.6%, Guizhou 25.4%, Chongqing 7.8%, Gansu 5.8%), and the recovery rate was 93.2%.

### 2.2. Study tools

A questionnaire was used as the survey instrument for this study, including General Information Questionnaire for elderly individuals, Activity of Daily Living Scale (ADL) to selected participants, the Walsh Family Resilience Questionnaire Chinese Version tested among elderly individuals, the Family Care Capacity Scale for Elderly Patients used as a concurrent validation tool for the caregivers, and the Psychological Resilience Scale and the Social Support Assessment used as concurrent validation tools for both the elderly individuals and the caregivers.

#### 2.2.1. General information questionnaire

The general information questionnaire includes sociodemographic and home care-related information for disabled elderly individuals who live at home, such as age, gender, marriage, education level, place of residence, and degree of self-care.

#### 2.2.2. Activity of daily living scale (ADL)

The ADL scale (Katz et al., 1963) includes 10 items that assess eating, dressing, washing, bowel control, urination control, toileting, walking on level ground, bed and chair transfer, going up and down stairs, and bathing. The total score the scale ranged from 0 to 100. Scores from 61 to 100 indicated mildly dependent, scores from 41 to 60 indicated moderately dependent, and scores from 0 to 40 indicated severely dependent.

#### 2.2.3. Chinese version of the Walsh Family Resilience Questionnaire (WFRQ-C)

The Walsh-FRQ was developed by Walsh (2016b) in 2016, corresponding to her family resilience process model. In this study, the Chinese version of the WFRQ-C (Wang and Lu, 2022), which was developed by our team, was used to evaluate disabled elderly individuals. After conducting a large-group reliability and validity study, there were 26 items across three factors: family beliefs, communication and resolution, and external support. Each item was scored on a 5-point Likert scale ranging from 1 (“never”) to 5 (“always”). Higher scores indicate higher levels of family resilience. The specific steps of translation, back-translation, comparison, language adaptation, and pilot testing can be found in the previous study on the WFRQ-C (Wang and Lu, 2022).

#### 2.2.4. Connor-Davidson Resilience Scale 10 (CD-RISC-10)

The 25-item Connor-Davidson Resilience Scale (CD-RISC), developed by Connor and Davidson (2003) in 2003 to quantify resilience and assess treatment response, is a widely used clinical tool with a very good psychometric rating. Campbell-Sills and Stein revised and improved this scale into the 10-item CD-RISC scale (CD-RISC-10) in 2007 (Campbell-Sills and Stein, 2007). This scale uses a 5-point scale ranging from 0 (“never”) to 4 (“almost always”). Higher scores indicate higher levels of resilience. In this study, Cronbach’s α coefficient for this scale among disabled elderly individuals and caregivers was 0.91 and 0.90, respectively.

#### 2.2.5. Family Caregiving Competence Scale for the Elderly (FCCSE)

This study adopted the Chinese version of the Family Care Capacity Scale for Elderly Patients (FCCSE) revised by Xiu et al. (2016), including 10 items across three factors: cognitive competence of family caregivers, family cohesion, and family support capability. Each item was scores on a 5-point Liker scale ranging from 1 to 5. The total score ranges from 10 to 50 points. A higher score indicates a stronger family care ability. In the current study, Cronbach’s α coefficient for this scale was 0.75.

#### 2.2.6. Social Support Rating Scale (SSRS-10)

The Social Support Rating Scale compiled by Shuiyuan (1994) was used to measure the extent to which individuals received psychological support in social life and the use of support. The scale contains 10 items across three factors: subjective support, objective support, and utilization of support. Higher scores indicate higher levels of social support. In the current study, Cronbach’s α coefficient for this scale among disabled elderly people and caregivers was 0.79 and 0.73, respectively.

### 2.3. Data collection and ethics consideration

The validation of this study started by conducting personal cognitive interviews with 20 community-dwelling elderly individuals based on the WFRQ-C. Then, formal investigation was performed. In the homes of the disabled elderly individuals or in the offices of community service center, the subjects (including disabled elderly individuals and primary caregivers) were asked to complete the WFRQ-C together and independently complete their own questionnaires. Disabled elderly individuals completed the CD-RISC-10 and SSRS-10, while caregivers completed the CD-RISC-10, FCCSE, and SSRS-10. Under normal circumstances, self-reports were used, but when the subjects could not complete the questionnaires due to educational level, visual impairment, disease or other reasons, the investigators read the questions one by one with a neutral and non-judgmental attitude, and the research subjects answered orally. All participants provided informed consent. The data were maintained securely, and only researchers had access to them. To compensate respondents for their time, a gift costing approximately 2 dollars was provided.

### 2.4. Statistical methods

**FIGURE 1:**
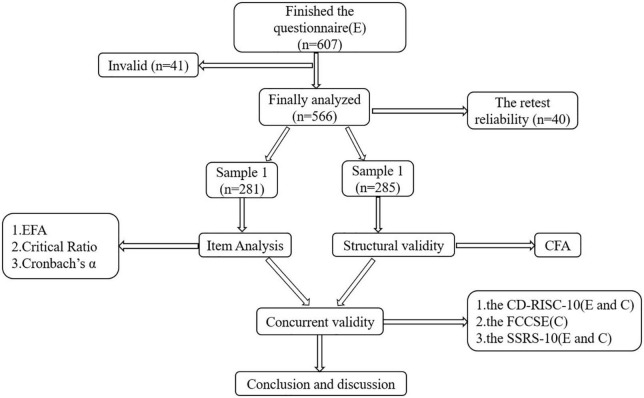
Validation process among community-dwelling disabled elderly individuals.

Statistical analysis was performed using SPSS 26.0 and AMOS 23.0 software. SPSS 26.0 software was used to randomly divide the total sample of disabled elderly individuals into two groups at a ratio of approximately 1:1 for analysis. For sample 1 (281), the item-total score Pearson correlation coefficient, item critical ratio value (CR value), EFA, Cronbach alpha coefficient, and Guttman split-half reliability coefficient analysis were used. For sample 2 (285), we used CFA. The total sample (566) was included to examine calibration-related validity. Test-retest reliability was calculated for sample 3 (40), which included participants who were retested after 2 weeks. A two-sided test level of *a* = 0.05 and *P* < 0.05 were considered statistically significant. The overall process can be seen in Figure 1.

#### 2.4.1. Item analysis

According to classical measurement theory (Nunnally, 1994), descriptive statistical analysis was carried out on the 26 items of the WFRQ-C, and the mean and standard deviation of each item were calculated to evaluate the distribution and variability of the items. The item-total score Pearson correlation coefficient was analysed to test the item representativeness. In the EFA to detect the number of factors and item loadings, it was first tested whether the KMO value was >0.7 and whether the *p* value of Bartlett’s/df was <0.05; if yes, it indicates that it is suitable for factor analysis. Then, the maximum variance method was used for principal component analysis. The number of extracted factors was based on eigenvalues > 1 and a higher factor load and percentage of explained variance than 0.4. The results were explained theoretically and empirically (Floyd and Widaman, 1995). When calculating the item CR value, the total family resilience score of all study subjects is arranged in order of high and low, the top 26% of the scores are in the high group, and the bottom 26% of the scores are in the low group. The significance level of the difference between the two was calculated by using two independent samples *T* tests. A significant CR value (*P* < 0.05) indicated that the item was able to identify the degree of response of different subjects, which was meaningful in the survey (Nunnally, 1994).

#### 2.4.2. Structural validity

Structural equation modeling was used for CFA to evaluate the fit of the factor structure proposed by EFA. The chi-square/freedom degree (χ2/df), Tucker–Lewis index (TLI), incremental fit index (IFI), comparative fit index (CFI), parsimony goodness-of-fit index (PGFI), and root-mean-square error of approximation (RMSEA) were selected to evaluate the fitting of the model. Bayesian corrected self-sampling was adopted (Zhao et al., 2010), the number of self-samplings was set to 2000, and the effect size and 95% confidence interval were estimated.

#### 2.4.3. Concurrent validity

The correlation between the total and each factor score of the Walsh Family Resilience Questionnaire among community-dwelling disabled elderly individuals (WFRQ-CE) and the CD-RISC-10, the FCCSE, and the SSRS-10 were analysed. This study assumes a positive correlation between family resilience scores and individual resilience, family care capacity, and social support.

#### 2.4.4. Reliability

Cronbach’s alpha coefficient for the total family resilience score and the scores of each factor were used to analyse the internal consistency, the Guttman split-half reliability coefficient was used to analyse the split-half reliability, and the intraclass correlation coefficient (ICC) was used to calculate the retest reliability of the total family resilience score and the scores of each factor of the 40 disabled elderly individuals who were retested after 2 weeks.

### 2.5. Ethical approval

This study was approved by the Institutional Review Board of Research Institutions (IRB). Participation was entirely voluntary, verbal and written consent were obtained prior to all interviews, and the informants’ anonymity and right to withdraw from the study prior to analysis were guaranteed. The interviews were conducted when the participants felt alert and ready. Contact information for psychological assistance was provided if needed. All participant information collected in the context of this study was anonymous and stored securely by the researchers.

## 3. Results

### 3.1. General information

The characteristics of disabled elderly individuals are shown in Table 1. The age of disabled elderly individuals ranged from 60 to 102 years old. Of the participants, 78.3% experienced mild dependence, 15.7% medium dependency, and 6.3% heavy dependency. There were no significant differences between the two randomly selected samples in terms of sociodemographic and family characteristics.

**TABLE 1:** Sociodemographic and family characteristics of participants.

Characteristics	Total (*N* = 566) $\bar{x}\pm S\,D$/*N* (%)	Sample 1 (*N* = 281) $\bar{x}\pm S\,D$/*N* (%)	Sample 2 (*N* = 285) $\bar{x}\pm S\,D$/*N* (%)	*t*/χ^2^	*p*
Age (year)	72.31 ± 8.13	72.66 ± 7.97	71.96 ± 8.28	1.025	0.31
**Gender**					
Male	270 (47.7)	140 (49.8)	130 (45.6)	1.004	0.32
Female	296 (52.3)	141 (50.2)	155 (54.4)		
**Ethnic**					
Han Chinese	526 (92.9)	262 (93.2)	264 (92.6)	0.079	0.78
Other	40 (7.1)	19 (6.8)	21 (7.4)		
**Religious**					
Yes	77 (13.6)	33 (11.7)	44 (15.4)	1.643	0.20
No	489 (86.4)	248 (88.3)	241 (84.6)		
**Marital status**					
Unmarried/divorced/separated	25 (4.4)	10 (3.6)	15 (5.3)	1.046	0.59
Married	400 (70.7)	199 (70.8)	201 (70.5)		
Widowed	141 (24.9)	72 (25.6)	69 (24.2)		
**Educational level**					
Junior high school and below	428 (75.6)	201 (71.5)	227 (79.6)	5.362	0.07
Senior high school	94 (16.6)	56 (19.9)	38 (13.3)		
College and above	44 (7.8)	24 (8.5)	20 (7.0)		
**Place of residence**					
City	223 (39.4)	121 (43.1)	102 (35.8)	3.446	0.18
Town	100 (17.7)	49 (17.4)	51 (17.9)		
Countryside	243 (42.9)	111 (39.5)	132 (46.3)		
**Living condition**					
Living with children	119 (21.0)	52 (18.5)	67 (23.5)	2.251	0.52
Living with a spouse	236 (41.7)	121 (43.1)	115 (40.4)		
With children and spouses and relatives and friends	141 (24.9)	71 (25.2)	70 (24.5)		
Living alone	70 (12.4)	37 (13.2)	33 (11.6)		
**Child counting**					
No one	9 (1.6)	3 (1.1)	6 (2.1)	3.017	0.56
1 child	117 (20.7)	65 (23.1)	52 (18.2)		
2 children	156 (27.6)	75 (26.7)	81 (28.4)		
3 children	135 (23.9)	64 (22.8)	71 (24.9)		
≥4 children	149 (26.3)	74 (26.3)	75 (26.3)		
**Per capita monthly income (¥)**					
<1000	113 (20.0)	54 (19.2)	59 (20.7)	6.232	0.10
1000–2999	156 (27.6)	69 (24.6)	87 (30.5)		
3000–4999	160 (28.3)	78 (27.8)	82 (28.8)		
5000 and above	137 (24.2)	80 (28.5)	57 (20.0)		
**Self-care level**					
Mild dependence	443 (78.3)	220 (78.3)	223 (78.2)	0.003	0.10
Medium dependency	89 (15.7)	44 (15.7)	45 (15.8)		
Heavy dependency	34 (6.0)	17 (6.0)	17 (6.0)		

### 3.2. Validity

#### 3.2.1. Item analysis and differentiation

The mean score of each item was 2.14–4.02, and the standard deviation was 0.86–1.35. Table 2 shows the details of this analysis. The item-total score Pearson correlation analysis results showed that each item was positively correlated with the total score; the correlation coefficients ranged from 0.185 to 0.739, and they were statistically significant (*P* < 0.01) (see Table 2). The scale items were analysed and screened using the CR value as the discriminant validity index between items. The independent sample *t* test results comparing the high and low groups showed that the statistical value of each item reached a significant level (*P* < 0.05).

**TABLE 2:** Walsh Family Resilience Questionnaire among community-dwelling disabled elderly individuals (WFRQ-CE) item-total score correlation analysis results and factor loading (*n* = 281).

Item	Item content	Average	SD	*r*	*F*_1_	*F*_2_	*F*_3_	*F*_4_
1	We face difficulties together as a team, rather than individually.	4.02	0.92	0.558**	**0.498**	0.470	0.140	−0.230
2	We view distress with our situation as common, understandable.	3.85	0.86	0.534**	**0.640**	0.274	0.102	−0.139
3	We approach a crisis as a challenge we can manage and master with shared efforts.	3.73	0.91	0.624**	**0.691**	0.267	0.175	−0.036
4	We try to make sense of our stressful situation and focus our options.	3.68	0.90	0.598**	**0.661**	0.102	0.211	0.214
5	We keep hopeful and confident that we will overcome our difficulties.	3.85	0.93	0.664**	**0.684**	0.232	0.328	−0.102
6	We focus on possibilities and we try to accept what we can’t change.	3.68	0.91	0.517**	**0.445**	0.360	0.063	0.115
7	We share important values and purpose that help us rise above difficulties.	3.43	1.07	0.700**	**0.477**	0.236	0.442	0.196
8	We draw on spiritual resources (religious or non-religious) to help us cope well.	2.14	1.35	0.185**	0.027	−0.120	0.020	**0.689**
9	Our challenges inspire creativity, more meaningful priorities, and stronger bonds.	3.33	1.00	0.535**	**0.575**	−0.155	0.392	0.270
10	Our hardship has increased our compassion and desire to help others.	3.78	0.95	0.531**	**0.648**	0.263	−0.004	0.088
11	We believe we can learn and become stronger from our challenges.	3.75	0.86	0.628**	**0.597**	0.309	0.192	0.087
12	Strong leadership from parents/caregivers provide warm nurturing, guidance, and security.	3.85	1.05	0.660**	0.231	**0.611**	0.357	−0.001
13	We can count on family members to help each other in difficulty.	3.99	0.98	0.640**	0.233	**0.709**	0.263	−0.103
14	In our immediate and extended family, we have positive role models and mentors.	3.61	1.03	0.646**	0.227	**0.639**	0.231	0.161
15	We can rely on support of friends and our community.	3.02	1.16	0.540**	0.065	0.350	0.236	**0.681**
16	We have economic security to be able to get through hard times.	3.71	1.09	0.569**	0.236	**0.613**	0.073	0.226
17	We can access community resources to help our family through difficult times.	2.69	1.24	0.519**	0.056	0.320	0.204	**0.731**
18	We try to clarify information about our stressful situation and options.	3.69	0.89	0.707**	0.371	**0.558**	0.283	0.183
19	We are clear and consistent in what we say and do.	3.78	0.96	0.679**	0.163	0.223	**0.813**	0.055
20	We can express our opinions and be truthful with each other.	3.80	1.01	0.689**	0.237	0.198	**0.772**	0.059
21	We can share difficult negative emotions (e.g., sadness, anger, fears).	3.44	1.07	0.529**	−0.006	0.083	**0.705**	0.299
22	We show each other understanding and avoid blame.	3.70	1.01	0.680**	0.150	0.245	**0.783**	0.093
23	We can share positive feelings, appreciation, humor, fun and find relief from difficulties.	3.78	0.99	0.673**	0.317	0.279	**0.626**	−0.002
24	We collaborate in discussing making decisions, and we handle disagreements fairly.	3.70	0.89	0.730**	0.335	0.417	**0.531**	0.093
25	We celebrate successes and learn from mistakes.	3.83	0.89	0.739**	0.428	0.409	**0.480**	0.068
26	We plan and prepare for the future and try to prevent crises.	3.53	1.07	0.647**	0.251	**0.565**	0.280	0.173

**Indicates that *P* < 0.01, F1, F2, F3, F4 are factors 1, 2, 3, 4.

#### 3.2.2. Structural validity

The results of EFA showed that the KMO value was 0.916 (KMO > 0.05), the Bartlett sphericity test value was 3702.894; these values were statistically significant (*P* < 0.001), and thus, factor analysis could be performed. Using the principal component analysis method, four common factors were extracted after orthogonal rotation by the maximum variance method (the eigenvalues of each common factor were all >1), and the cumulative explained variance was 56.94%. The factor loading of each item is shown in Table 2.

**FIGURE 2:**
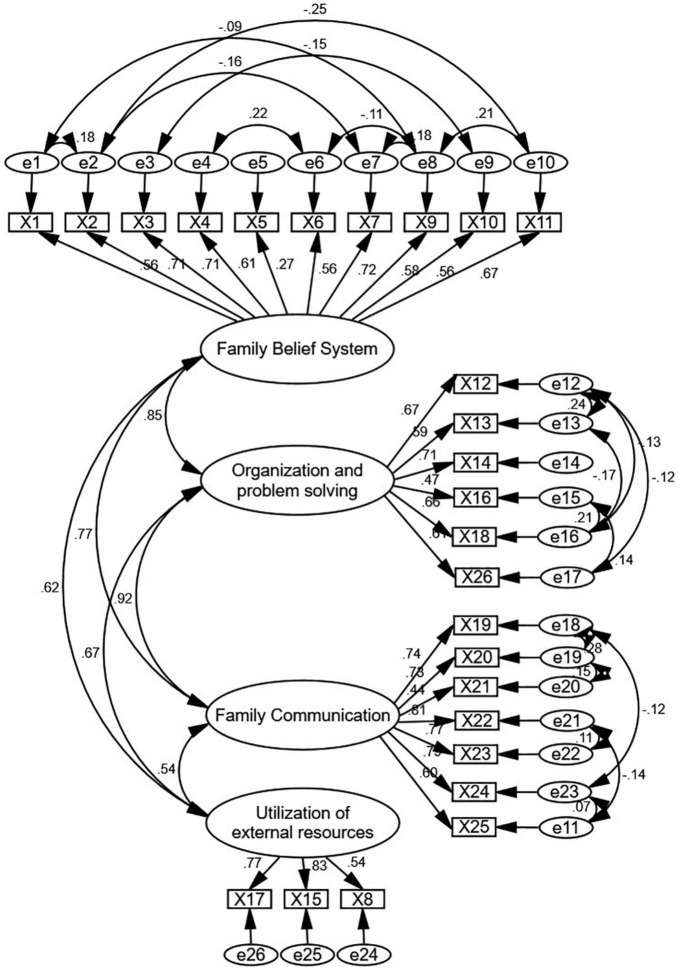
Standardized four-factor structural equation model of Walsh Family Resilience Questionnaire among community-dwelling disabled elderly individuals (WFRQ-CE).

The CFA used AMOS 23.0 software to construct a 4-factor structural equation model. The fit indices for this model were as follows: χ2 = 548.043, χ2/df = 2.007 < 5, *P* < 0.001, TLI = 0.900, IFI = 0.917, CFI = 0.916, RMSEA = 0.06, and PGFI = 0.681. These indices indicated that the model fit was acceptable. The standardized regression coefficients ranged from 0.27 to 0.83, as shown in Figure 2.

#### 3.2.3. Concurrent validity

Using the CD-RISC-10, FCTI-25, FCCSE, and SSRS-10 as the efficacy criteria, the overall sample was tested for the correlation validity of the criteria. The results showed that the total score of the scale and its factors were significantly correlated with each criterion questionnaire. The results are shown in Table 3.

**TABLE 3:** The correlation between each factor of the Walsh Family Resilience Questionnaire among community-dwelling disabled elderly individuals (WFRQ-CE) and the calibration variables.

Calibration variables	$\bar{x}\pm S\,D$	Family beliefs	Organization and problem solving	Family communication	Utilization of external resources	Total score
CD-RISC-10 (E).	24.59 ± 7.19	0.47**	0.48**	0.47**	0.31**	0.54**
CD-RISC-10 (C).	27.79 ± 5.92	0.55**	0.58**	0.51**	0.28**	0.60**
FCCSE (C)	33.22 ± 4.94	0.25**	0.28**	0.25**	0.44**	0.35**
SSRS-10 (E)	36.65 ± 7.72	0.27**	0.35**	0.27**	0.23**	0.35**
SSRS-10 (C)	41.82 ± 6.90	0.23**	0.44**	0.45**	0.50**	0.46**

**Indicates that *P* < 0.01.

E, disabled elderly individuals; C, caregivers; CD-RISC-10, Connor-Davidson Resilience Scale 10; FCCSE, Family Care-giving Competence Scale for the Elderly; SSRS-10, Social Support Rating Scale.

### 3.3. Reliability

### 3.3.1. Internal consistency confidence

Cronbach’s α coefficient for the WFRQ-CE total scale was 0.93, and Cronbach’s α coefficients for the four factors (i.e., family belief, organization and problem solving, family communication, and utilization of external resources) was 0.87, 0.83, 0.89, and 0.65, respectively.

### 3.3.2. Retest reliability

The retest reliability of the total scale was 0.96, and the retest reliability of the four factors (i.e., family belief, organization and problem solving, family communication, and utilization of external resources) was 0.95, 0.92, 0.92, and 0.95, respectively.

### 3.3.3. Split-half reliability

The split-half reliability of the WFRQ-CE total scale was 0.85, and the split-half reliability of the four factors (i.e., family belief, organization and problem solving, family communication, and utilization of external resources) was 0.81, 0.78, 0.79, and 0.68, respectively.

## 4. Discussion

### 4.1. Items of WFRQ-CE are suitable

From a statistical point of view, the Pearson correlation coefficients of the item-total score of WFRQ-CE ranged from 0.50 to 0.80 except for item 8 (*r* = 0.183). When the high and low groupings were taken as 26% before and after, the CR values of the items reached a significant level (*P* < 0.05). If the standard deviation of item 8 > 1, the discrimination is acceptable. There was no ceiling effect for each item (the first and last options were selected >50%) (Jingnan et al., 2021). The factor analysis indicated that the factor loading for item 8 was 0.689 (>0.4), which indicated an acceptable level of representativeness (Hao and Jingwu, 2006). The 26 items on the WFRQ-CE are reserved; this indicates that all 26 items on the WFRQ-C are suitable for community-dwelling disabled elderly individuals in China.

### 4.2. The WFRQ-CE is reliable and further validated among community elders

Reliability, also known as accuracy, reflects the measurement results stability of a research tool (Hao and Jingwu, 2006). Cronbach’s α coefficients above 0.7 are generally considered acceptable, and the Cronbach’s α coefficients for subscales are preferably above 0.6. The stability of the scale is better when the split-half and retest confidences are above 0.7 (Hao and Jingwu, 2006; Minglong, 2010). The reliability coefficients of WFRQ-CE are all within the acceptable range, indicating that the WFRQ-CE exhibits good reliability.

Validity refers to the closeness of the measurement results of the research tool to the target content, reflecting the authenticity and accuracy of the research tool (Hao and Jingwu, 2006). Walsh pointed out that the nine core processes of the family resilience theoretical framework are recursive, and the key processes have synergistic effects, which can reasonably be clustered in different ways in different environments and populations (Walsh, 2016a). In this study, the EFA yielded four common factors with eigenvalues > 1, and the cumulative variance contribution rate was 56.94%. Compared with the general community residents in China (Wang and Lu, 2022), the item attribution is the same as that of the family belief and external support domains but the factor of “communication and resolution” in the community resident group. The results of the test for disabled elderly individuals divided this part into two factors, namely, “organization and problem solving” and “family communication” in this study. This is consistent with the model of coping with “disability” in families of the elderly in the qualitative study, where problem solving and communication processes are relatively independent (Abulaiti et al., 2022). Compared with the three domains and nine processes of the Walsh family resilience model developed by the original scale (Walsh, 2016a), the items included in F1 (family belief) in this study are all derived from its family belief system items. The items included in F2 (organization and problem solving) involve family organizational processes and communication and problem-solving processes, which mainly emphasize the core process of family flexible coping and problem solving. F_3_ (Family Communication) contains items that are communication/problem solving processes and focuses on the expression of ideas and emotions between families. F_4_ (utilization of external resources) contains items related to family belief systems and family organization processes that measure the support of religion, community, and friends. Each of the adaptive indicators in the adjusted CFA is within a reasonable range, indicating that the internal structure of the scale is relatively stable in the families of disabled elderly individuals. The factor structure and item levels of the WFRQ-CE were different from those of the WFRQ-IT (Rocchi et al., 2017), which was used in families with chronic childhood diseases. They mainly indicated that family belief and communication had interaction. This may be related to different sociocultural environments and groups. This is similar to the distinction between solving somatic problems and psychological communication for the elderly (Hsu, 2009). Family resilience may be understood in unique ways across families, but the common metric helps to understand variations across contexts.

The correlation coefficient between the tested scale and the calibration scale should be between 0.4 and 0.8 (Hao and Jingwu, 2006). Previous research has found that characteristics of individual resilience such as intrinsic control trajectories, emotional regulation, belief systems, and self-efficacy are protective factors that promote family resilience at the individual level (Meihua, 2013). Families have large social networks, and the more resources to which they have access, the stronger their resilience and the more easily they can adjust the family model and division of human resources flexibly to suit the situation, which allowed the family to adapt to adversity. The type of resources could be physical, financial, natural, traditional, social capital, and human resources (Yang et al., 2021). In this study, the CD-RISC-10, FCCSE, and SSRS-10 were used as the efficacy criteria. The correlations between these scales and the WFRQ-CE total scale ranged from 0.35 to 0.60, which indicates a moderately positive correlation between family resilience and personal resilience, caring ability, and social support in families of disabled elderly individuals. Moreover, family resilience includes other elements, which is the important theoretical basis for families’ ability to cope and adapt to adversity (Walsh, 2002). These findings also show that the WFRQ-CE has a suitable concurrent validity, which contains the primary source of adaptive capacity for the elderly and their caregivers in the face of disabling adversity.

## 5. Limitations

Although this study underwent multicenter data collection and rigorous data testing, there were some limitations. First, those with serious physical diseases or extreme weakness were excluded because they were not able to complete the questionnaire, which may impact the representativeness of the study sample to some extent. Second, the types of disability included in this study do not include dementia groups, and attention should be given to identifying the scope of use.

## 6. Conclusion

The results of this study show that the WFRQ-CE has good reliability and validity in disabled elderly individuals population in China, including four factors: family belief, organization and problem solving, family communication, and utilization of external resources. The WFRQ-CE can be used as a stable, reliable, concise, and effective tool for measuring family resilience in community-dwelling disabled elderly individuals. The application of this tool in longitudinal and intervention studies should be further considered.

## Data availability statement

The raw data supporting the conclusions of this article will be made available by the authors, without undue reservation.

## Ethics statement

The studies involving human participants were reviewed and approved by Fudan University Ethics Committee. The patients/participants provided their written informed consent to participate in this study.

## Author contributions

All authors listed have made a substantial, direct, and intellectual contribution to the work, and approved it for publication.

## Footnotes

1
http://www.cmw-gov.cn/news.view-794-1.html
